# Obesity and Mechanical Thrombectomy

**DOI:** 10.7759/cureus.12671

**Published:** 2021-01-13

**Authors:** David R Hallan

**Affiliations:** 1 Neurosurgery, Penn State Health Milton S. Hershey Medical Center, Hershey, USA

**Keywords:** neurosurgery, stroke, thrombectomy, obesity, outcomes, mortality, hemorrhagic conversion, trinetx, bmi, cerebrovascular

## Abstract

Background

Obesity has been shown to have a positive mortality benefit in patients undergoing percutaneous coronary intervention, dialysis, those with rheumatoid arthritis, chronic obstructive pulmonary disease, and various wasting diseases. Studies for this mortality benefit in ischemic stroke patients are conflicting, and it has not been well studied in mechanical thrombectomy patients. We sought to determine the impact of obesity on outcomes of mechanical thrombectomy patients.

Methodology

We used a large global health research network to gather clinical data extracted from the electronic medical records of ischemic stroke patients who underwent mechanical thrombectomy, and then stratified these patients into obese and non-obese cohorts. The primary endpoint was mortality.

Results

After propensity score matching, obese patients who underwent mechanical thrombectomy had decreased mortality (p = 0.0033, odds ratio = 0.81, 95% confidence interval = 0.704,0.932) compared to non-obese patients. No statistically significant difference was shown between these two cohorts for the outcomes of ventilator dependence, hemicraniectomy, or post-procedure intracerebral hemorrhage.

Conclusion

Despite increasing risk of ischemic stroke, obese patients who undergo mechanical thrombectomy have decreased mortality rates compared to their non-obese counterparts.

## Introduction

Obesity increases the risk of stroke [1-4]. Risk increases roughly 6% with increase in body mass index (BMI) [4]. Despite this, obesity has been shown to have a positive mortality benefit in stroke patients [4-14]; however, not all studies agree [15,16]. A recent study examined mortality as an outcome for ischemic stroke patients undergoing mechanical thrombectomy. The study found that higher BMI is associated with decreased intracerebral hemorrhage post-procedure, and that BMI positively correlates with non-hemorrhagic inpatient mortality [3]. We sought to support or refute this claim using a multi-institutional database.

## Materials and methods

The TriNetX research database was retrospectively queried to evaluate all patients with a diagnosis of ischemic stroke who underwent mechanical thrombectomy. The patients were then divided into cohorts of obese and overweight versus non-obese and non-overweight patients according to the International Classification of Diseases, Tenth Revision code E66. Analysis was performed using unmatched and propensity score-matched cohorts using known stroke risk factors. The primary endpoint was mortality. The secondary endpoints included ventilator dependence, hemicraniectomy, and intracerebral hemorrhage. Hazard ratios were calculated using R Studio’s survival package v3.2-3 and were validated comparing the output to that of SAS version 9.4. Chi-square analysis was performed on categorical variables.

## Results

The baseline demographics and characteristics are shown in Table 1. Of the patients who underwent mechanical thrombectomy, 3,230 were obese and 8,256 were non-obese.

**Table 1:** Baseline demographics and characteristics. Top box represents cohort 1: thrombectomy and obese. Bottom box represents cohort 2: thrombectomy and non-obese. ICD, International Classification of Diseases, Tenth Revision; Index, date of thrombectomy; Max, maximum; Min, minimum; SD, standard deviation; Std diff, standard difference

Demographics, ICD 10 codes/Diagnoses	Mean ± SD	Min	Max	P-Value	Std diff	Patients	% of Cohort
Age at index	67.2 ± 13.4	13	90	<0.0001	0.2372	3,230	100%
	70.5 ± 14.6	0	90			8,256	100%
				<0.0001	0.105	1,804	56%
Female						4,179	51%
				<0.0001	0.105	1,426	44%
Male						4,077	49%
				<0.0001	0.1512	2,382	74%
Unknown race						6,613	80%
				<0.0001	0.0902	652	20%
White						1,378	17%
				<0.0001	0.1456	189	6%
African American						238	3%
				0.8821	0.0031	10	0%
Asian						27	0%
E08-E13				<0.0001	0.4556	1,602	50%
Diabetes mellitus						2,308	28%
E78				<0.0001	0.4244	2,398	74%
Disorders of lipoprotein metabolism and other lipidemias						4,487	54%
I10-I16				<0.0001	0.4455	2,749	85%
Hypertensive diseases						5,489	66%
I20-I25				<0.0001	0.2162	1,424	44%
Ischemic heart diseases						2,775	34%
I73				<0.0001	0.1782	573	18%
Other peripheral vascular diseases						947	11%
J40-J47				<0.0001	0.2939	1,250	39%
Chronic lower respiratory diseases						2,076	25%
F17				<0.0001	0.1478	704	22%
Nicotine dependence						1,323	16%
F10.1				0.0958	0.0339	126	4%
Alcohol abuse						270	3%
N17-N19				<0.0001	0.2712	1,143	35%
Acute kidney failure and chronic kidney disease						1,912	23%
K74				<0.0001	0.0836	73	2%
Fibrosis and cirrhosis of liver						97	1%
I48				<0.0001	0.1062	1,188	37%
Atrial fibrillation and flutter						2,621	32%
I50				<0.0001	0.2549	1,127	35%
Heart failure						1,932	23%

**Figure 1:**
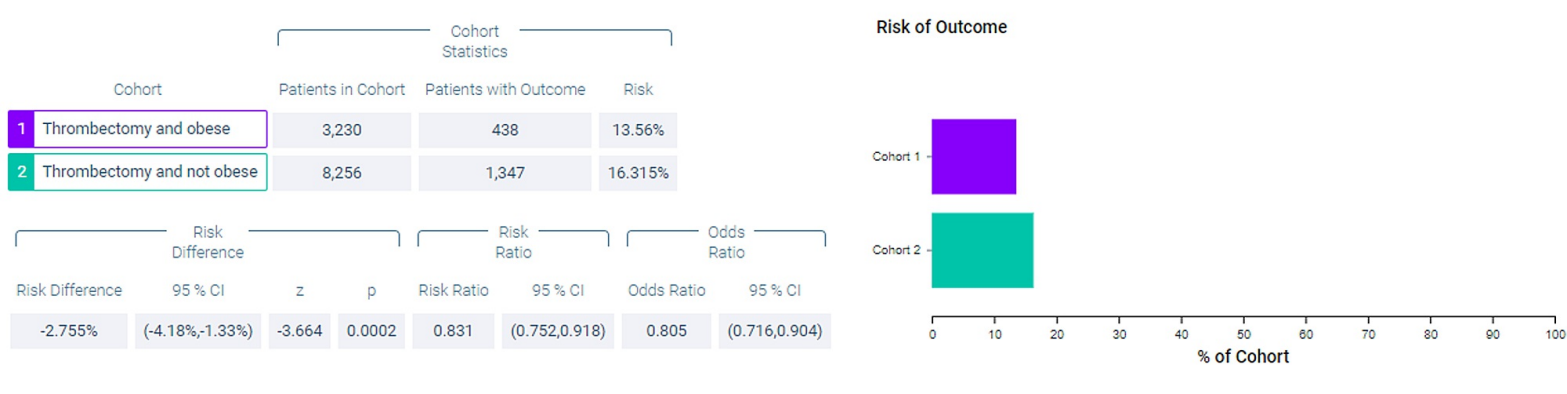
Measures of association for the outcome deceased.

**Figure 2:**
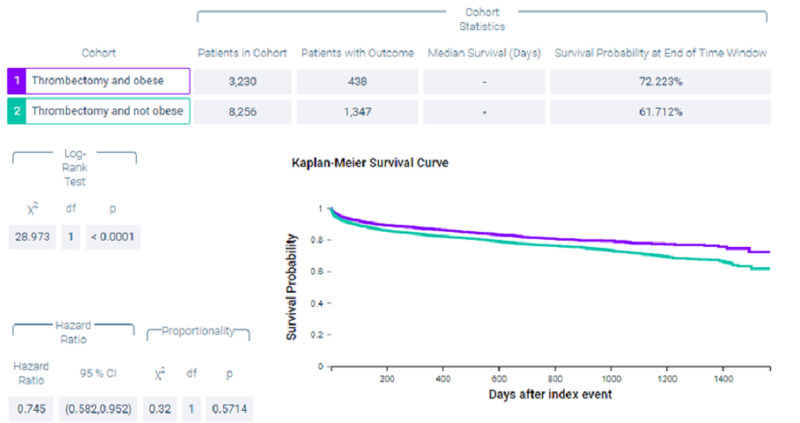
Kaplan-Meier analysis for the outcome deceased. Index event, date of thrombectomy

Figure 1 shows measures of association for cohort 1 (thrombectomy and obese) versus cohort 2 (thrombectomy and non-obese) for the outcome of mortality. Figure 2 shows a Kaplan-Meier analysis for this outcome. A total of 13.56% of patients in cohort 1 and 16.315% of patients in cohort 2 died (p < 0.0002, odds ratio [OR] = 0.805, 95% confidence interval [CI] = 0.716,0.904). Survival probability at the end of 1,600 days after mechanical thrombectomy was 72.223% for cohort 1 and 61.712% for cohort 2.

**Figure 3:**
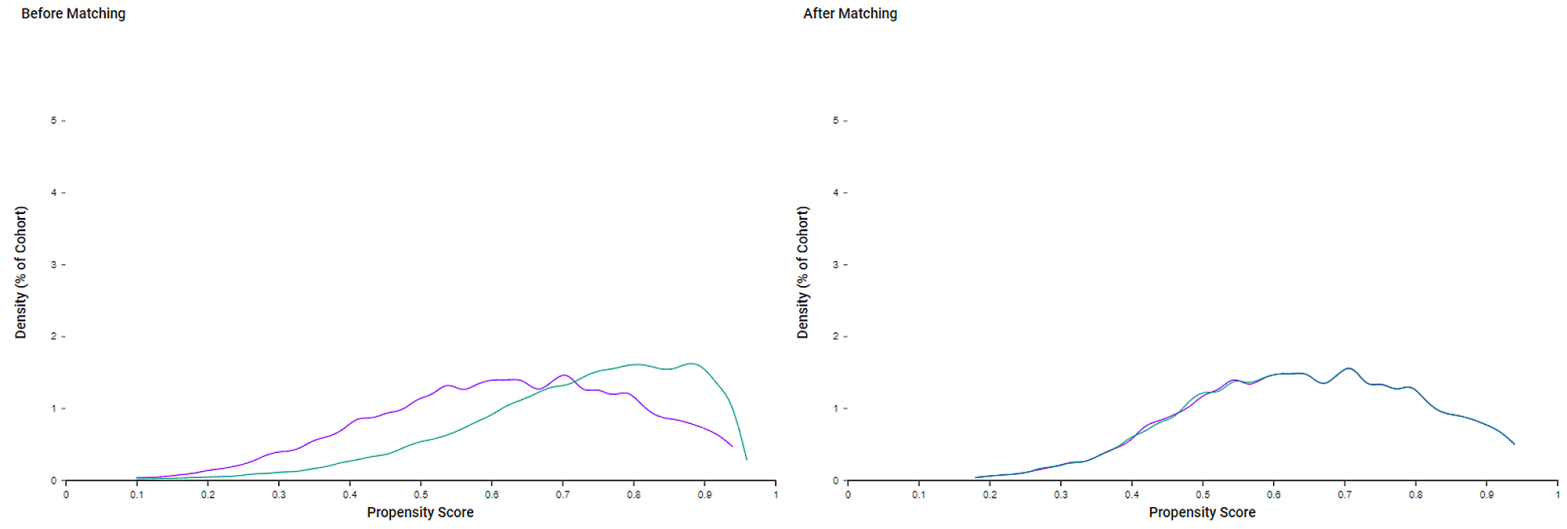
Propensity score matching. Purple: thrombectomy and obese. Green: thrombectomy and non-obese.

Because of the differences in baseline demographics seen in Table 1, cohorts were propensity score-matched (Figure 3). As seen in Table 2, after propensity score matching, both cohort 1 (thrombectomy and obese) and cohort 2 (thrombectomy and non-obese) included 3,020 patients.

**Table 2:** Baseline demographics and characteristics after matching. Top box represents cohort 1: thrombectomy and obese. Bottom box represents cohort 2: thrombectomy and non-obese. ICD, International Classification of Diseases, Tenth Revision; Index, date of thrombectomy; Max, maximum; Min, minimum; SD, standard deviation; Std diff, standard difference

Demographics, ICD 10 codes/Diagnoses	Mean ± SD	Min	Max	P-Value	Std diff	Patients	% of Cohort
Age at index	67.9 ± 13.1			0.8706	0.0042	3,020	100%
	67.9 ± 15.3					3,020	100%
				0.9056	0.0031	2,258	74.77%
Female						2,262	74.90%
				0.7959	0.0067	1,656	54.83%
Male						1,666	55.17%
				0.7959	0.0067	1,364	45.17%
Unknown race						1,354	44.83%
				0.6753	0.0108	608	20.13%
White						595	19.70%
				0.5562	0.0151	147	4.87%
African American						157	5.20%
				1	<0.0001	10	0.33%
Asian						10	0.33%
E08-E13				0.6002	0.0135	2,539	84.07%
Diabetes mellitus						2,524	83.58%
E78				0.885	0.0037	2,198	72.78%
Disorders of lipoprotein metabolism and other lipidemias						2,203	72.95%
I10-I16				0.8165	0.006	1,411	46.72%
Hypertensive diseases						1,420	47.02%
I20-I25				0.3353	0.0248	1,298	42.98%
Ischemic heart diseases						1,261	41.76%
I73				0.7687	0.0076	1,106	36.62%
Other peripheral vascular diseases						1,095	36.26%
J40-J47				0.6878	0.0103	1,098	36.36%
Chronic lower respiratory diseases						1,083	35.86%
F17				0.228	0.031	1,014	33.58%
Nicotine dependence						970	32.12%
F10.1				0.7634	0.0077	1,002	33.18%
Alcohol abuse						991	32.82%
N17-N19				0.7741	0.0074	623	20.63%
Acute kidney failure and chronic kidney disease						614	20.33%
K74				0.6279	0.0125	507	16.79%
Fibrosis and cirrhosis of liver						493	16.33%
I48				0.2357	0.0305	115	3.81%
Atrial fibrillation and flutter						98	3.25%
I50				0.846	0.005	53	1.76%
Heart failure						55	1.82%

**Figure 4:**
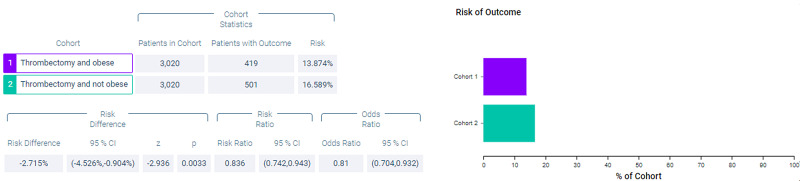
Measures of association for the matched cohort; outcome: deceased.

**Figure 5:**
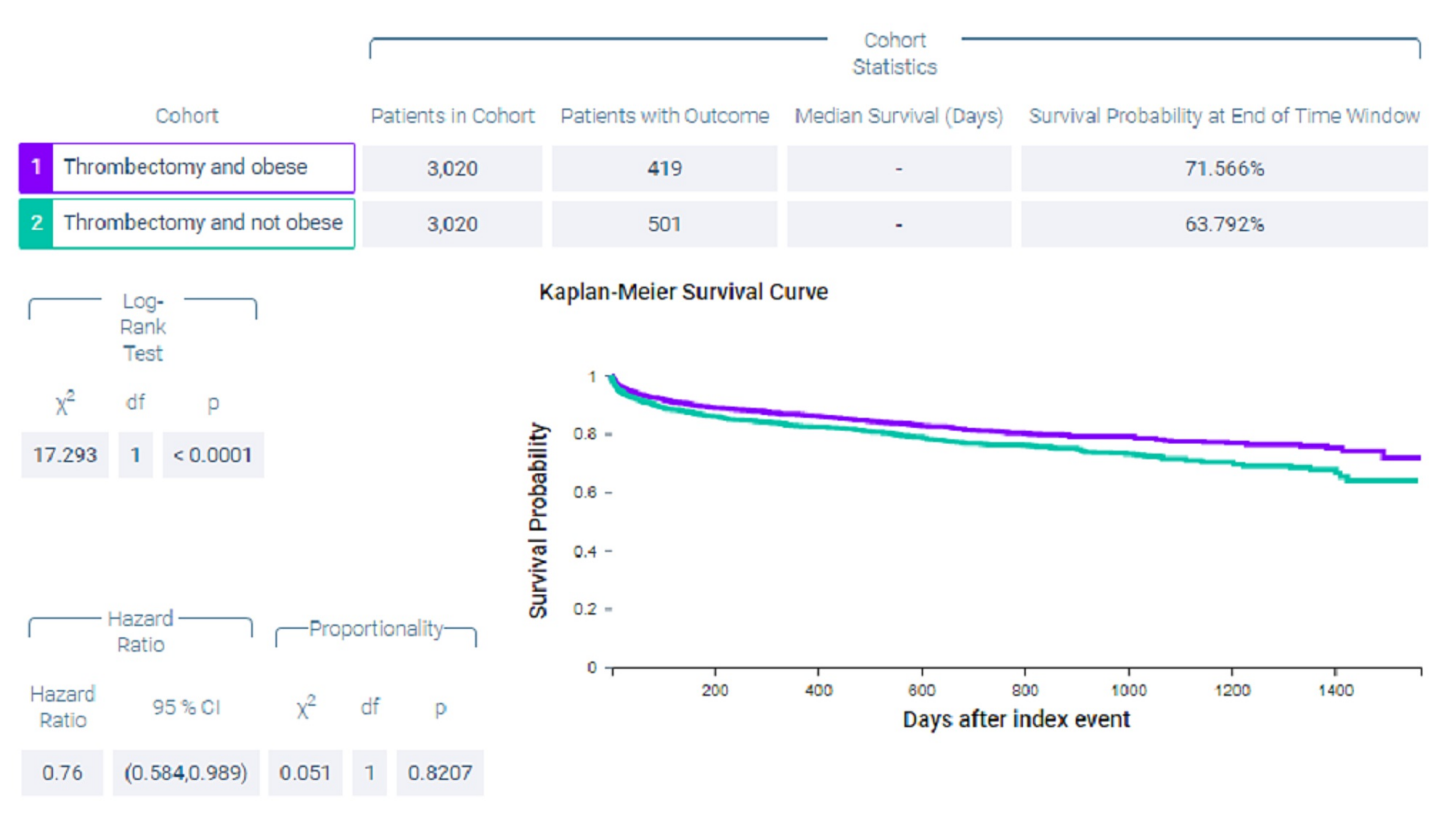
Kaplan-Meier analysis for the matched cohort; outcome: deceased.

Figure 4 shows measures of association for both cohort 1 (thrombectomy and obese) and cohort 2 (thrombectomy and non-obese) for the outcome of mortality. Figure 5 shows a Kaplan-Meier analysis for this outcome. A total of 13.874% of patients in cohort 1 and 16.589% of patients in cohort 2 died (p = 0.0033, OR = 0.81, 95% CI = 0.704,0.932). Survival probability at the end of 1,600 days after mechanical thrombectomy was 71.566% for cohort 1 and 63.792% for cohort 2.

**Figure 6:**
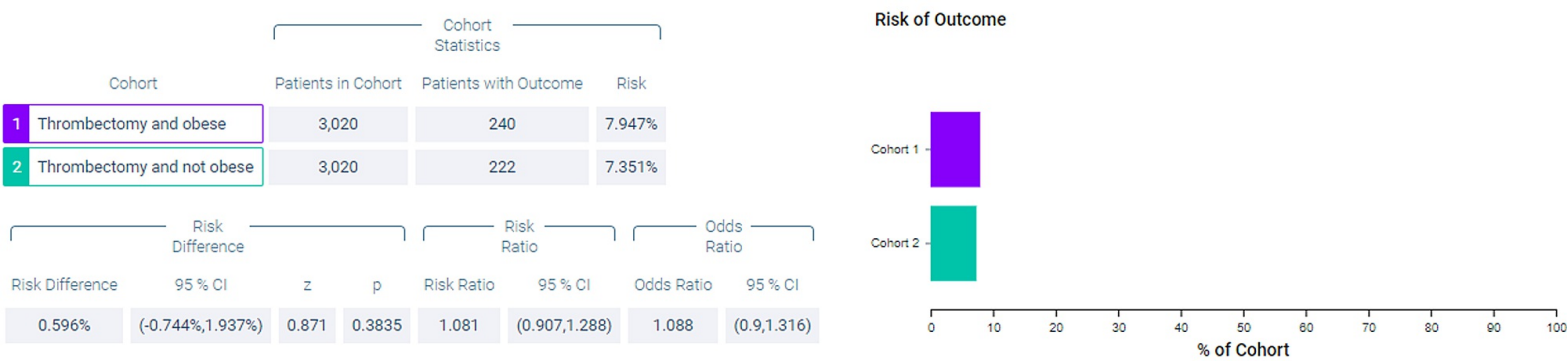
Measures of association for the matched cohort; outcome: ventilator dependence.

**Figure 7:**
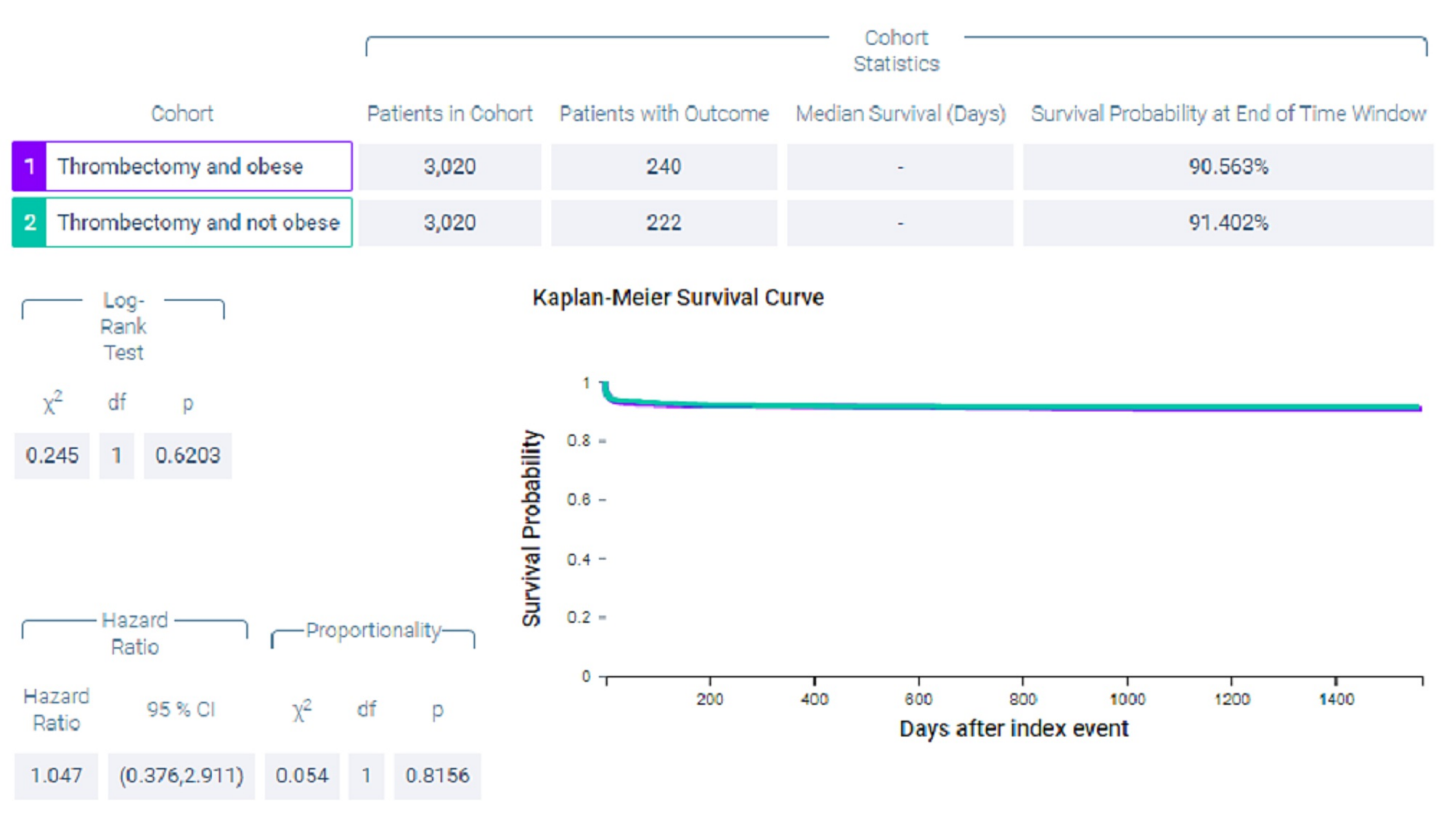
Kaplan-Meier analysis for the matched cohort; outcome: ventilator dependence.

Figure 6 shows measures of association for both cohort 1 (thrombectomy and obese) and cohort 2 (thrombectomy and non-obese) for the outcome of ventilator dependence. Figure 7 shows a Kaplan-Meier analysis for this outcome. A total of 7.947% of patients in cohort 1 required ventilator use compared to 7.351% of patients in cohort 2 (p = 0.3835, OR = 1.088, 95% CI = 0.9,1.316).

**Figure 8:**
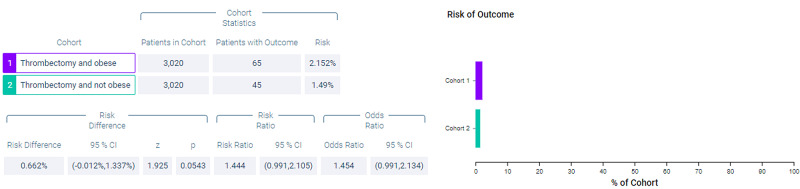
Measures of association for the matched cohort; outcome: hemicraniectomy.

**Figure 9:**
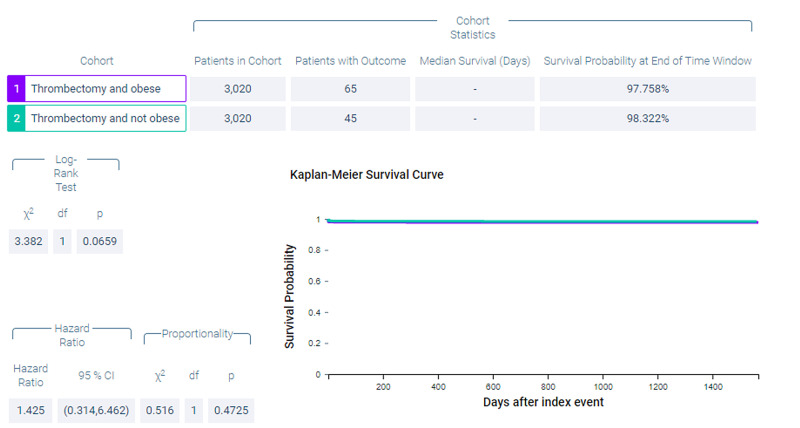
Kaplan-Meier analysis for the matched cohort; outcome: hemicraniectomy.

Figure 8 shows measures of association for both cohort 1 (thrombectomy and obese) and cohort 2 (thrombectomy and non-obese) for the outcome of hemicraniectomy. Figure 9 shows a Kaplan-Meier analysis for this outcome. A total of 2.152% of patients in cohort 1 underwent hemicraniectomy compared to 1.49% of patients in cohort 2 (p = 0.0543, OR = 1.454, 95% CI = 0.991,2.134).

**Figure 10:**
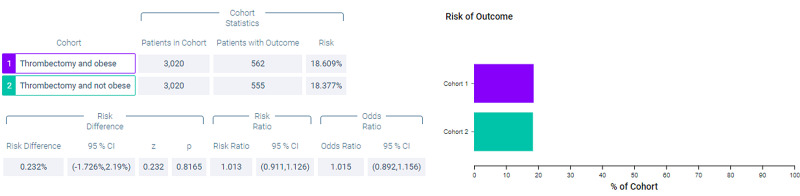
Measures of association for the matched cohort; outcome: intracerebral hemorrhage.

**Figure 11:**
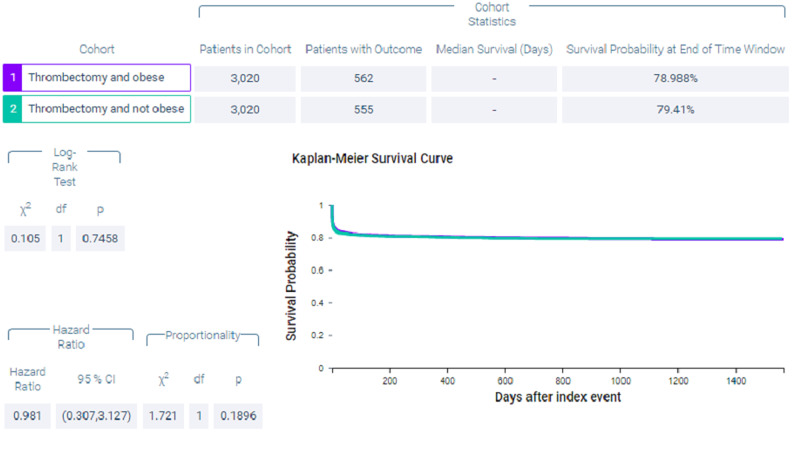
Kaplan-Meier analysis for the matched cohort; outcome: intracerebral hemorrhage.

Figure 10 shows measures of association for both cohort 1 (thrombectomy and obese) and cohort 2 (thrombectomy and non-obese) for the outcome of intracerebral hemorrhage. Figure 11 shows a Kaplan-Meier analysis for this outcome. A total of 18.609% of patients in cohort 1 experienced post-procedural intracerebral hemorrhage compared to 18.377% of patients in cohort 2 (p = 0.8165, OR = 1.015, 95% CI = 0.892,1.156).

## Discussion

Over 30% of the world’s population is estimated to be overweight, and this number is increasing [4]. A meta-analysis from 2008 showed that patients with higher BMI had better mortality in heart failure. Further studies have shown similar outcomes in patients undergoing percutaneous coronary intervention, dialysis, those with rheumatoid arthritis, chronic obstructive pulmonary disease, and various wasting diseases [4,17-22]. This became known as the obesity paradox, wherein those undergoing vascular reperfusion for myocardial infarction and obese patients had decreased mortality [3,23,24].

Of the 12 studies examining BMI and stroke, 10 showed higher BMI to be associated with lower mortality [4-14]. Two of the studies did not show such an association when adjusted for stroke severity [16] and when looking at mortality within a 30-day period [15]. To my knowledge, only one study examined BMI as it relates to mechanical thrombectomy. It is possible that higher BMI might influence time to arterial puncture due to increased difficulties gaining arterial access in obese patients, transporting obese patients, and transferring obese patients [3]. They found that higher BMI is associated with decreased intracerebral hemorrhage post-procedure, and that BMI correlates with higher non-hemorrhagic inpatient mortality [3]. However, the results of this study disagree and show no statistical difference in post-procedure hemorrhage rates, as well as decreased mortality in obese patients.

This analysis was not without limitations. The major limitation of this study was that it was retrospective in nature. Furthermore, due to the nature of the database, patient-level data on specific outcomes could not be collected. Moreover, radiology information was not available. Additionally, information on the type of diagnostic test used for the confirmation of disease was not available. The data collected was for billing purposes, not for clinical use, and thus much clinical information is missing. In addition, some misidentification is inevitable in database studies.

## Conclusions

Despite increasing risk of ischemic stroke, obese patients who undergo mechanical thrombectomy have decreased mortality rates compared to their non-obese counterparts. Future studies can use a frailty index to assess mortality in this population.
